# Development Trends of White Matter Connectivity in the First Years of Life

**DOI:** 10.1371/journal.pone.0024678

**Published:** 2011-09-23

**Authors:** Pew-Thian Yap, Yong Fan, Yasheng Chen, John H. Gilmore, Weili Lin, Dinggang Shen

**Affiliations:** 1 Department of Radiology and Biomedical Research Imaging Center, University of North Carolina, Chapel Hill, North Carolina, United States of America; 2 Department of Psychiatry, University of North Carolina, Chapel Hill, North Carolina, United States of America; Beijing Normal University, China

## Abstract

The human brain is organized into a collection of interacting networks with specialized functions to support various cognitive functions. Recent research has reached a consensus that the brain manifests small-world topology, which implicates both global and local efficiency at minimal wiring costs, and also modular organization, which indicates functional segregation and specialization. However, the important questions of how and when the small-world topology and modular organization come into existence remain largely unanswered. Taking a graph theoretic approach, we attempt to shed light on this matter by an in vivo study, using diffusion tensor imaging based fiber tractography, on 39 healthy pediatric subjects with longitudinal data collected at average ages of 2 weeks, 1 year, and 2 years. Our results indicate that the small-world architecture exists at birth with efficiency that increases in later stages of development. In addition, we found that the networks are broad scale in nature, signifying the existence of pivotal connection hubs and resilience of the brain network to random and targeted attacks. We also observed, with development, that the brain network seems to evolve progressively from a local, predominantly proximity based, connectivity pattern to a more distributed, predominantly functional based, connectivity pattern. These observations suggest that the brain in the early years of life has relatively efficient systems that may solve similar information processing problems, but in divergent ways.

## Introduction

The human brain is a complex system that is capable of integrating massive amount of information with startling efficiency. A comprehensive description of the architecture of the anatomical connectivity patterns is therefore fundamentally important in cognitive neuroscience and neuropsychology, as it reveals how functional brain states emerge from their underlying structural substrates and provides new mechanistic insights into the association of brain functional deficits with the underlying structural disruption [1].

Principled means of assessing early brain development contribute positively to assessing mental health in fetuses and neonates. On T1-weighted images, however, most white matter in the neonatal brain is unmyelinated and therefore exhibits lower intensity than gray matter. This ambiguous image contrast, in addition to the dynamic change of image appearance caused by rapid myelination in the first year of life, confounds analysis of brain growth in this essential period of development. Diffusion tensor imaging (DTI), on the other hand, yields a different kind of contrast that is based on the characterization of water diffusion patterns and allows more straightforward characterization of developing white matter fiber tracts. The fractional anisotropy of white matter fibers, for instance, increases with age, reflecting increasing organization and myelination. The application of DTI to the examination of neonatal brain development can therefore provide valuable information on the neurodevelopmental origins of psychiatric illness [2]. Structural brain changes associated with psychosis and other major psychiatric illnesses are thought to develop early during fetal or neonatal life [3].

Although there has been a great deal of recent interest in the study of childhood and adolescent brain development, very little is known about the brain network in the first years of life, which is perhaps the most dynamic phase of postnatal brain development. The current study is the first attempt to characterize brain growth in this period of life using network graphs with connectivity quantified using DTI fiber tractography. We compute various measures, such as global and local efficiency [4]–[6], of the backbone brain connectivity network, observe how these measures change with growth, and compare them with comparable regular and lattice networks. We seek to validate whether the pediatric brain exhibits the small-world property commonly observed in previous studies on adult subjects [7]–[14]. We corroborate our findings with measures, such as fiber length, to add another dimension of validation for our observations. We also study the topology of the brain network by modularity [15], [16] based separation of the connection nodes into different communities. We observe how these communities evolve with time and infer pertinent physiological implications. This report, while confirming many findings from previous studies, sheds new light on the developmental mechanism of the human brain.

## Materials and Methods

### Data Acquisition and Post-Processing

This study involved 39 subjects (18 males, 21 females) in three age groups: 2-week-olds (gestational age, mean 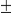 SD: 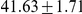 weeks), 1-year-olds (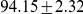 weeks), and 2-year-olds (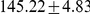 weeks). This dataset was provided to us by Dr. John Gilmore of the University of North Carolina from his neonatal project on early brain development [17]. Informed written consent was obtained from the parents and the experimental protocols were approved by the Institutional Review Board of the University of North Carolina (UNC) School of Medicine. None of the subjects was sedated for MRI. Before the subjects were imaged, they were fed and fitted with ear protection. The neonates were swaddled. All subjects slept during the imaging examination. For each subject, diffusion-weighted images were acquired using a Siemens 3T head-only scanner (Allegra; Siemens Medical System) at 2 weeks, 1 year, and 2 years. Diffusion gradients with a 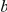-value of 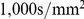 were applied in six non-collinear directions, 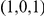, 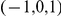, 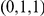, 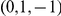, 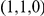, and 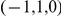. A 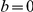 reference scan was also acquired. Forty-five contiguous slices with a slice thickness of 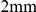 covered a field of view (FOV) of 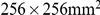 with an isotropic voxel size of 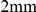. Ten acquisitions were used to improve the signal-to-noise ratio (SNR) in the images. The acquisition typically takes 6.5 minutes. A weighted least squares estimation method was used to construct the diffusion tensors [18], [19]. All images were visually inspected before analysis to ensure no bulk motion.

### Spatial Normalization

**Figure 1:**
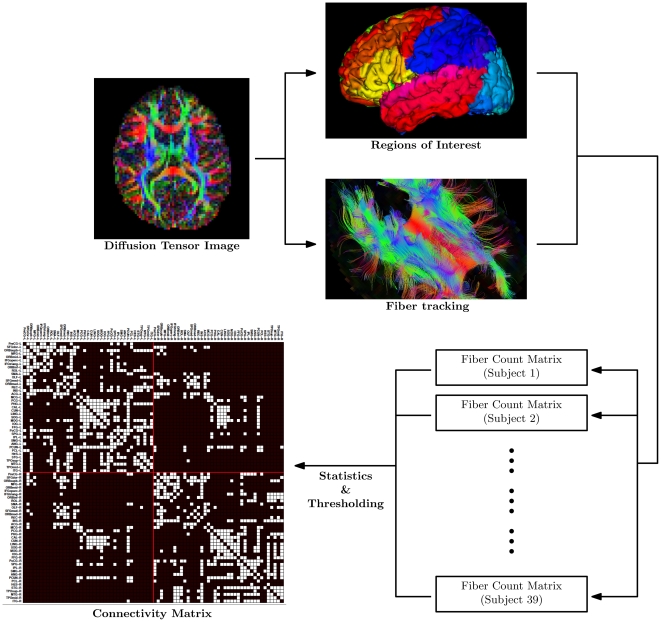
Obtaining the Connectivity Matrix. A schematic digram illustrating the major processes involved in generating the final connectivity maps. Streamline fiber tractography was performed on each diffusion tensor image and a connectivity matrix was computed based on the AAL [23] ROIs. The fiber count matrices were constructed by enumerating the number of fibers connecting each region pair. The connectivity matrix, indicating consistent connections, was generated by thresholding the fiber count statistics.

For each subject, the images at the 2-week and 1-year time points were registered to the image at the 2-year time point using the DTI non-rigid registration algorithm described in [20], [21]. The DTI registration algorithm uses regional tensor distribution information and tensor edge information to hierarchically guide registration of a pair of DT images. To leverage longitudinal information for more accurate spatial alignment, we first estimated the correspondences of the 2-week-time-point images with respect to their 1-year-time-point counterparts. Correspondences to the 2-year-time-point images were then estimated by concatenating the deformation fields with those of the 1-year-time-point images with respect to the 2-year-time-point images. Correspondences of all the 2-year-time-point images with respect to a template (i.e., the 2-year-time-point DT image of a randomly selected subject) were then determined, allowing all images from the different age groups to be analyzed based on a common stereotaxic space. The estimated deformation fields were stored for use in subsequent processing steps (see Fig. 1).

### Fiber Tractography

Whole-brain streamline fiber tractography [22] was then performed on each DT image in its native space with minimal seed point FA of 0.2, minimal allowed FA of 0.1, maximal turning angle of 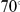, minimal fiber length of 20 mm and maximal fiber length of 200 mm. The motivation for a low FA value allowance was so that unmyelinated white matter fibers could be extracted. The fibers were warped to the common stereotaxic space using the deformation fields determined as described in the previous section, allowing us to correct for factors such as brain size and inter-subject spatial variation.

### Brain Parcellation

The Automated Anatomical Labeling (AAL) template [23] was co-registered to the template DT image to parcellate the brain space into 78 cortical regions (39 for each hemisphere; subcortical and cerebellar regions excluded; see Table 1 for details). We note that each region mask is not a pure cortical GM mask but includes tissues from both cortical GM and subcortical WM. The latter allowed us to determine which fibers were linked to a specific cortical region.

**Table 1:** Regions of Interest Based on the Automated Anatomical Labeling (AAL) Template.

Region	Abbrev	Region	Abbrev
Left Precentral Gyrus	PreCG-L	Right Precentral Gyrus	PreCG- R
Left Superior Frontal Gyrus (dorsal)	SFGdor -L	Right Superior Frontal Gyrus (dorsal)	SFGdor -R
Left Orbitofrontal Cortex (superior)	ORBsupb-L	Right Orbitofrontal Cortex (superior)	ORBsupb-R
Left Middle Frontal Gyrus	MFG-L	Right Middle Frontal Gyrus	MFG-R
Left Orbitofrontal Cortex (middle)	ORBmid-L	Right Orbitofrontal Cortex (middle)	ORBmid-R
Left Inferior Frontal Gyrus (opercular)	IFGoperc-L	Right Inferior Frontal Gyrus (opercular)	IFGoperc-R
Left Inferior Frontal Gyrus (triangular)	IFGtriang-L	Right Inferior Frontal Gyrus (triangular)	IFGtriang-R
Left Orbitofrontal Cortex (inferior)	ORBinf-L	Right Orbitofrontal Cortex (inferior)	ORBinf-R
Left Rolandic Operculum	ROL-L	Right Rolandic Operculum	ROL-R
Left Supplementary Motor Area	SMA-L	Right Supplementary Motor Area	SMA-R
Left Olfactory	OLF-L	Right Olfactory	OLF-R
Left Superior Frontal Gyrus (medial)	SFGmed-L	Right Superior Frontal Gyrus (medial)	SFGmed-R
Left Orbitofrontal Cortex (medial)	ORBmed-L	Right Orbitofrontal Cortex (medial)	ORBmed-R
Left Rectus Gyrus	REC-L	Right Rectus Gyrus	REC-R
Left Insula	INS-L	Right Insula	INS-R
Left Anterior Cingulate Gyrus	ACG-L	Right Anterior Cingulate Gyrus	ACG-R
Left Middle Cingulate Gyrus	MCG-L	Right Middle Cingulate Gyrus	MCG-R
Left Posterior Cingulate Gyrus	PCG-L	Right Posterior Cingulate Gyrus	PCG-R
Left ParaHippocampal Gyrus	PHG-L	Right ParaHippocampal Gyrus	PHG-R
Left Calcarine Cortex	CAL-L	Right Calcarine Cortex	CAL-R
Left Cuneus	CUN-L	Right Cuneus	CUN-R
Left Lingual Gyrus	LING-L	Right Lingual Gyrus	LING-R
Left Superior Occipital Gyrus	SOG-L	Right Superior Occipital Gyrus	SOG-R
Left Middle Occipital Gyrus	MOG-L	Right Middle Occipital Gyrus	MOG-R
Left Inferior Occipital Gyrus	IOG-L	Right Inferior Occipital Gyrus	IOG-R
Left Fusiform Gyrus	FFG-L	Right Fusiform Gyrus	FFG-R
Left Postcentral Gyrus	PoCG-L	Right Postcentral Gyrus	PoCG-R
Left Superior Parietal Gyrus	SPG-L	Right Superior Parietal Gyrus	SPG-R
Left Inferior Parietal Lobule	IPL-L	Right Inferior Parietal Lobule	IPL-R
Left Supramarginal Gyrus	SMG-L	Right SupraMarginal Gyrus	SMG-R
Left Angular Gyrus	ANG-L	Right Angular Gyrus	ANG-R
Left Precuneus	PCUN-L	Right Precuneus	PCUN-R
Left Paracentral Lobule	PCL-L	Right Paracentral Lobule	PCL-R
Left Heschl Gyrus	HES-L	Right Heschl Gyrus	HES-R
Left Superior Temporal Gyrus	STG-L	Right Superior Temporal Gyrus	STG-R
Left Temporal Pole (superior)	TPOsup-L	Right Temporal Pole (superior)	TPOsup-R
Left Middle Temporal Gyrus lef t	MTG-L	Right Middle Temporal Gyrus	MTG-R
Left Temporal Pole (middle)	TPOmid-L	Right Temporal Pole (middle)	TPOmid-R
Left Inferior Temporal Gyrus	ITG-L Right	Inferior Temporal Gyrus	ITG-R

### Connectivity

Two regions were considered as anatomically connected if fibers passing through their respective masks were present. For each subject, the number of fibers passing through every pair of regions was counted. These fiber counts were however taken as only an indication of the existence, and not weight, of an anatomical connection. For analyzing the brain network topology, we took a classical unweighted approach [4], [5], [24], since it was not obvious how the edge weights, i.e., the number of connection fibers, should be interpreted when computing the minimum path length [24].

**Figure 2:**
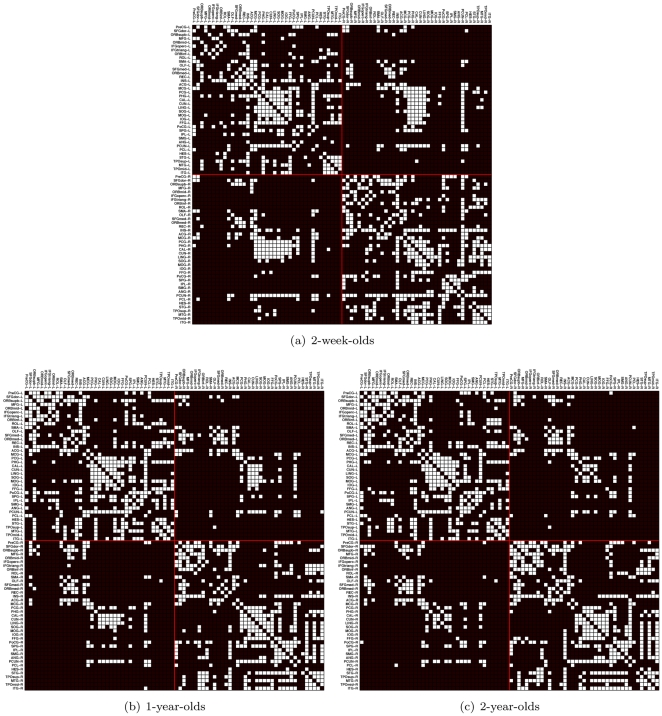
Connectograms. Connectivity matrices characterizing the backbone connections. The network cost is 0.21, which ensures that all nodes were full connected (see Fig. 3).

**Figure 3:**
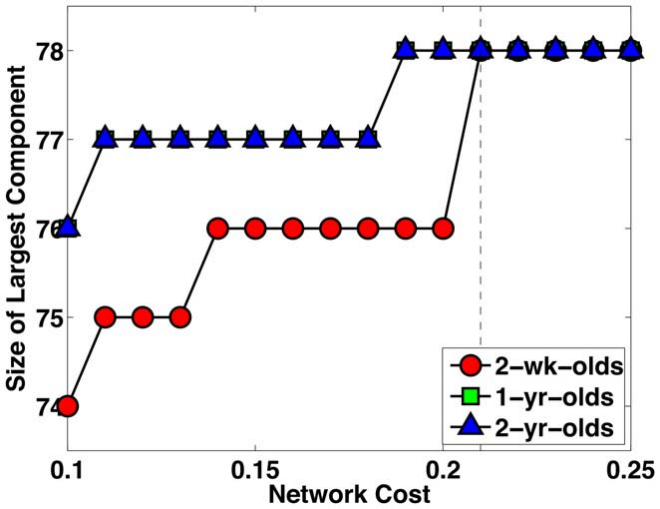
Largest Connected Component. The number of nodes of the largest connected component in all networks stabilize and reach the maximum possible value (78) at the network cost of 0.21, a value which we used for our analysis, unless otherwise stated.

Given the variability of brain anatomy, it is not surprising that anatomical connectivity between regions differs across subjects. In this study, we focused on the connections that were most consistent across subjects, i.e., the backbone network [13], [25]. To identify highly consistent connections, we computed the reciprocal of the coefficient of variation (i.e., the ratio of the mean to the standard deviation) of the fiber count for each pair of regions (total: 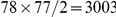 region pairs). In line with the convention of the signal processing society, we call this measure the signal-to-noise ratio (SNR). This connectivity SNR measure 1) is independent of the total number of fibers reconstructed during tractography (DT images of neonates tend to produce less fibers) and 2) caters for the fact that different regions might be connected with different numbers of fibers. Intuitively, a region pair that is connected with a consistent number of fibers is considered to exhibit high connectivity. We thresholded the resulting SNR matrix with a threshold that gave us a connectivity matrix with a specific network cost, indicating the degree of connection sparsity. However, considering that different thresholds would affect the number of connections in the resulting brain networks, we performed our analysis by applying different network costs. See Fig. 2 for an illustration of the connectivity matrices. The connectivity matrices are generated with a network cost of 0.21, where all the nodes are fully connected, as shown in Fig. 3. A fully connected network has no isolated nodes.

Fiber length information was also collected at the same time by taking the points on a fiber closest to the respective centroids of the connected regions as endpoints of a fiber segment. The average length of the connecting fibers were recorded for each pair of regions. Note that all fiber lengths were computed in the stereotaxic space to allow comparison across age groups in a common frame of reference.

### Network Metrics

Representing a network as an unweighted graph 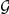 with 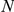 nodes, its metrics for global and local efficiency can be computed as [5], [6]

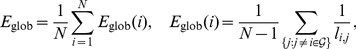
(1)

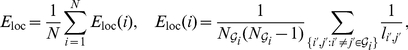
(2)where 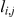 is the shortest path length between nodes 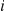 and 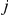, 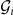 is a subgraph comprising nodes directly connected to node 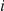, and 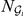 is the number of nodes, or degree of connections, of 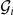. 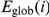 and 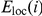 are nodal efficiency metrics. Specifically, 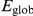 measures the efficiency of parallel information transfer in the network, whereas 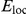 measures the local efficiency of information transfer in the immediate neighborhood of each node.

To measure how expensive it is to construct a network [6], we computed the cost of the network, defined as the total number of edges in a graph, divided by the maximum possible number of edges 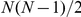:
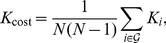
(3)where 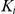 is the degree of each node 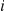, i.e., the number of nodes in subgraph 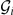.

A module of 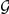 is a subset of nodes which are more densely connected to each other in the same module than to nodes outsides the module. For a configuration of modular organization 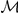 with 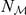 modules, its modularity 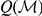 is defined as [15]

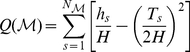
(4)where 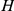 is the total number of edges in 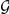, 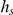 is the total number of edges in module 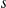, and 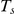 is the sum of the degrees of the nodes in module 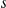. The modularity of a graph is defined as the largest value of modularity measures associated with all possible configurations of modules, which can be found by optimization algorithms [15]. We adopted a fast modularity optimization algorithm [16], which has been demonstrated to be capable of achieving solutions with quality comparable to existing algorithms, including simulated annealing [26], [27].

Topological roles of nodes in terms of their intra-modular and inter-modular connectivity patterns can be quantified by the normalized intra-modular degree and the participation coefficient [26]. The normalized intra-modular degree 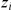 measures how dense a node 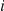 connects to other nodes in the same module, and the participation coefficient 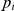 measures how a node 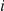 connects to nodes in other modules. Denoting the module to which node 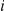 belongs as 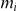, the normalized intra-modular degree and the participation coefficient are defined respectively as [26]

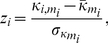
(5)

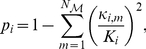
(6)where 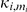 is the number of edges connecting the 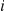-th node to other nodes in its module 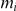, referred to as the intra-modular node degree; 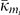 and 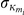 are the mean and standard deviation of intra-modular node degrees of all nodes in module 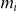, 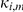 is the number of edges of the 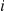-th node to 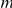-th module, and 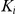 is the number of edges that connect node 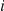 to all other nodes, i.e., the degree of node 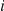.

Betweenness is a measure of the centrality of a node in a network. It is calculated as the fraction of shortest paths between node pairs that pass through the node of interest. Betweenness, in some sense, measures the influence of a node over the spread of information throughout the network [28]. The betweenness centrality [29] of a node 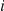 is defined as:
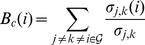
(7)where 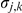 is the number of shortest paths from node 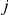 to 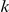, and 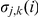 is the number of shortest paths that traverse node 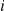. The quantity is normalized by 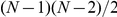 so that the greatest possible value is 1.

To evaluate the vulnerability [30] of the network to the damage of a particular node, we remove a node and its connections from the network and computed the change in network global efficiency:
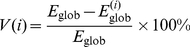
(8)where 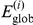 is the global efficiency after removing node 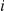. In most cases, a node with high betweenness value will typically also have a high vulnerability value, since its damage will cause a high degree of disruption of information flow.

### Permutation Test

To investigate the significance of the observed differences given by a specific network metric between brains of two different age groups, a permutation test were performed. First, the difference in the measured metric values between the two groups was calculated: this is the observed value of the test statistic. Then the samples in both groups were pooled. Next, the difference in the metric value was calculated and recorded for every permutation of labels of these pooled samples, while maintaining the original group sizes. The two-sided 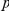-value of the test was then calculated as the proportion of sampled permutations where the absolute difference was greater than or equal to the absolute value of the observed value of the test statistic. 1000 permutations were performed for each test.

## Results

### Pediatic Brain Networks Have Small-World Topology

The brain networks were studied and compared with comparable random networks and regular lattices over multiple network costs in terms of their global and local efficiency [4]–[6]. We required the random networks to have not only the same number of nodes and edges, as proposed in [24], but also the same degree distribution as the brain networks in concern. This was achieved with the rewiring technique described in [31]. Preserving the degree distribution allows us to rule this factor out from the set of possible reasons of observed differences between the brain networks and the respective random networks.

**Figure 4:**
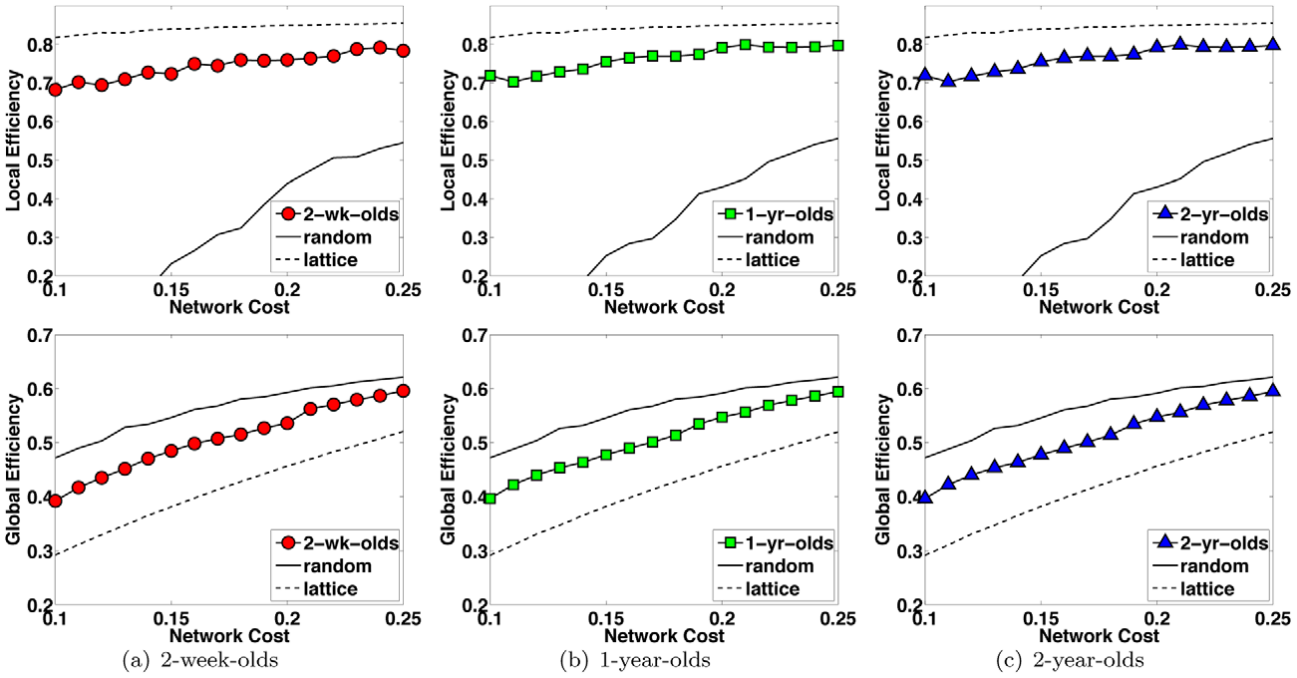
Network Efficiency. Local and global efficiency of pediatric brain networks of (a) 2-weeks-olds, (b) 1-year-olds and (c) 2-year-olds. All networks exhibit small-world nature, which is characterized by local efficiency greater than comparable random networks, and global efficiency greater than regular lattices [5], [6]. There is a general trend of efficiency increase with age. The neonatal brain network shows significantly lower efficiency compared to the other two age groups.

The top panels of Fig. 4 shows that the brain networks of all age groups have local efficiency higher than the equivalent random networks. This indicates that the networks are highly clustered or cliquish, conferring a capability of specialized or modular processing in local neighborhoods [32]–[34]. On the other hand, the bottom panels of Fig. 4 indicates that all networks consistently exhibit global efficiency higher than equivalent lattices, signifying efficient distributed and integrated processing over the entire network.

**Figure 5:**
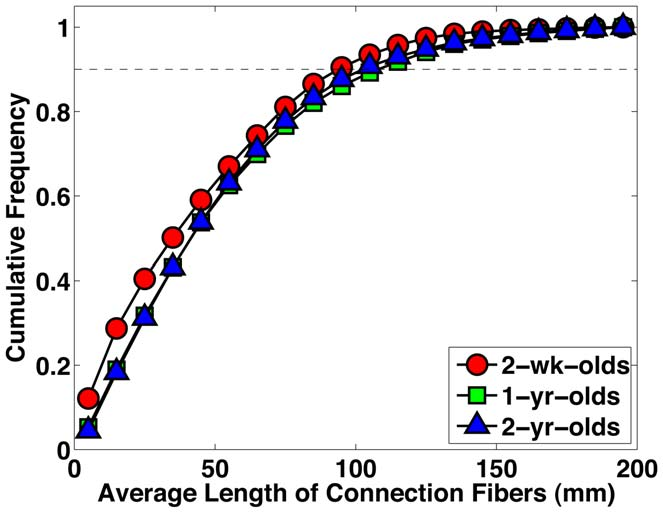
Inter-Region Connection Fiber Length Distribution. Cumulative distribution plots of the inter-region connection fiber lengths indicate that there is a progressive maturation of long fibers with growth. The dashed horizontal line marks the 0.90 frequency point and indicates that only a small fraction of the fibers are long fibers.

The two observations above indicate that the pediatric brain networks exhibit small-world topology – a good compromise between full connectivity, which would be very costly in terms of wiring and power supply, and a lattice topology, which impairs massively long distance communication. Fig. 5 shows that a vast majority of the connection fibers lies in the short end of the length spectrum, with only a small fraction accounting for longer connections. This observation is in agreement with the results of other recent studies on brain organization that suggest the brain favors locally dense communication and minimizes the number of long distance connections [35]. From the same figure, we can also observe that longer connections, in general, increase with growth. Table 2 shows that the difference in average fiber length (measured in the common space) between the 2-week-olds and the older age groups is statistically significant. This hints that there is a *local-to-distributed-organization* growth trend in early developing brains as suggested for more matured brains in [36], [37].

**Table 2:** Average Lengths of Connection Fibers (mm).

Age Group	2-week-olds	1-year-olds	2-year-olds
Fiber Length	40.22	66.16	62.45
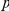 -value (w.r.t 2-week-olds)	-	<0.001	<0.001
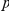 -value (w.r.t 1-year-olds)	<0.001	-	0.993
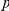 -value (w.r.t 2-year-olds)	<0.001	0.993	-

### Efficiency Increases With Growth

Unlike adult brains which are relatively stable structurally and functionally, pediatric brains undergo rapid changes. The first year of life is perhaps the most dynamic phase of postnatal brain development, with rapid development of a wide range of cognitive and motor functions [38]. From the point of view of brain topological network, the neonatal brain has lower local efficiency (permutation test, 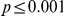), but similar global efficiency (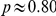), compared with that of 1-year-olds and 2-year-olds. This observation parallels the fact that myelination happens rapidly in the first year of life and begins to stabilize at the age of two. Myelination has a direct impact on the impulse propagation speed along the fiber, and the state of progressive myelination in the first year of life implies that many connections are in progress, and hence the overall lower efficiency.

### Pediatic Brain Networks Exhibit Broad-Scale Characteristic

**Figure 6:**
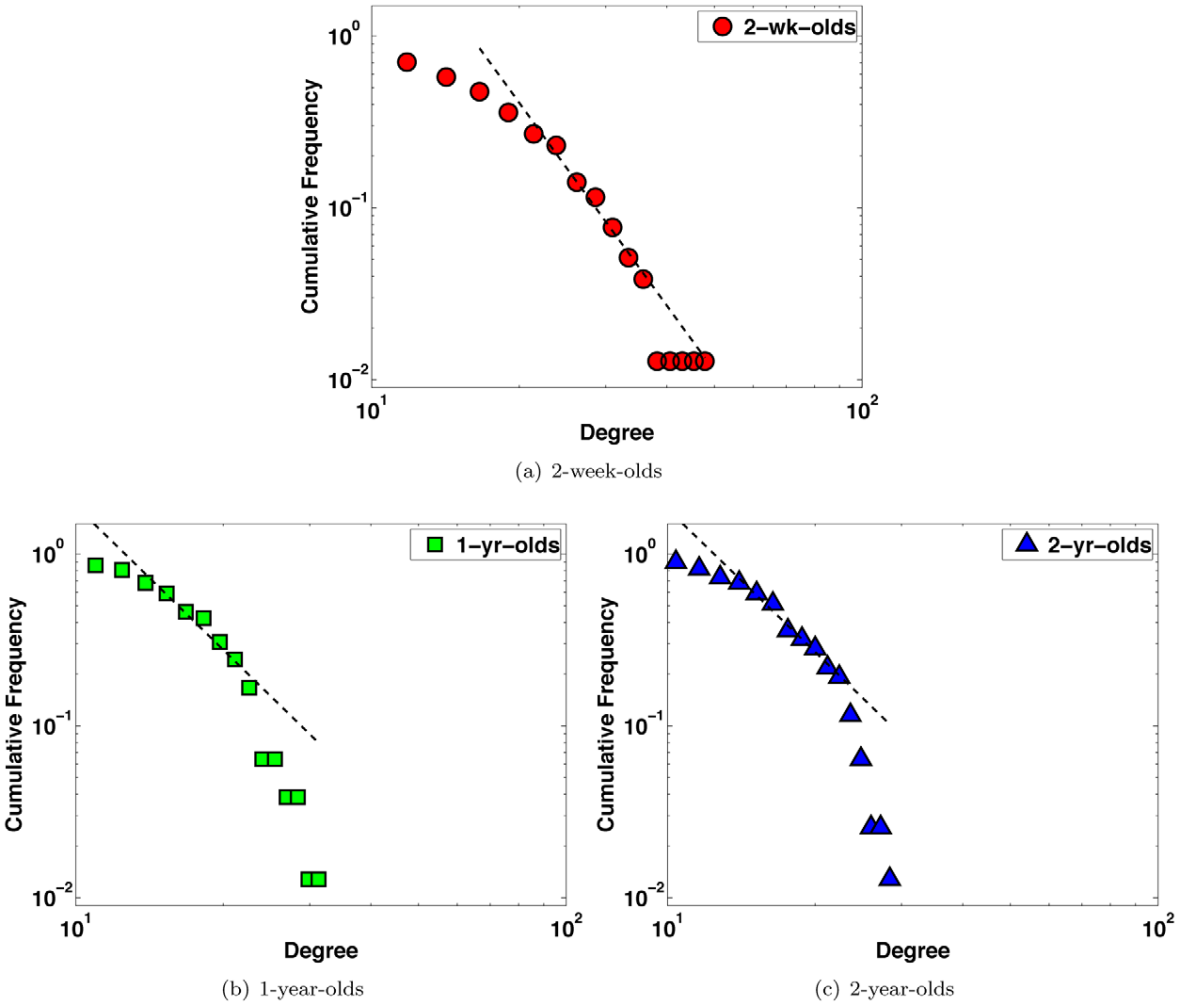
Node Degree Distributions. Single-scale, scale-free and broad-scale [39] are characterized by Gaussian/exponential decay, power law decay, and truncated power law decay, respectively. The node degree distributions give good indication that the pediatric brain networks are broad-scale in nature. In the double logarithmic plots, the degree distribution decays linearly before a sharp cutoff. The gradient magnitudes of the fitted lines are 3.921, 2.784 and 2.764 for (a), (b) and (c), respectively.

There are three classes of small-world networks [39]: (a) scale-free networks, characterized by a nodal connectivity distribution that decays as a power law; (b) broad-scale networks, characterized by a connectivity distribution that has a power law regime followed by a sharp cutoff; and (c) single-scale networks, characterized by a connectivity distribution with a fast (Gaussian or exponential) decaying tail. Each network has different degree of resilience to targeted attacks [8], [40]. We examined the node degree distributions of the early developing brain networks and found that they observed a truncated power law. The networks were hence broad-scale in nature. This is shown in Fig. 6, where we used cumulative distributions to reduce the effects of noise [41]. Goodness-of-fit of the straight line in the double logarithmic plot was tested using the coefficient of determination 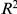 (better fit indicated by a value closer to 1), and the values given by the curves of the 2-week-olds, 1-year-olds, and 2-year-olds were 0.9791, 0.9495, and 0.9475, respectively. Fitting a function 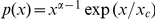 [13], [39] to the degree cumulative distribution gave 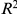 values of 0.9919, 0.9401, and 0.9475, respectively, again validating that the degree distributions observed the truncated power law, which is characteristic of broad-scale networks.

### The Pediatric Brain Networks Have Nonrandom Modularity and Exhibit Local-to-Distributed Organization

**Figure 7:**
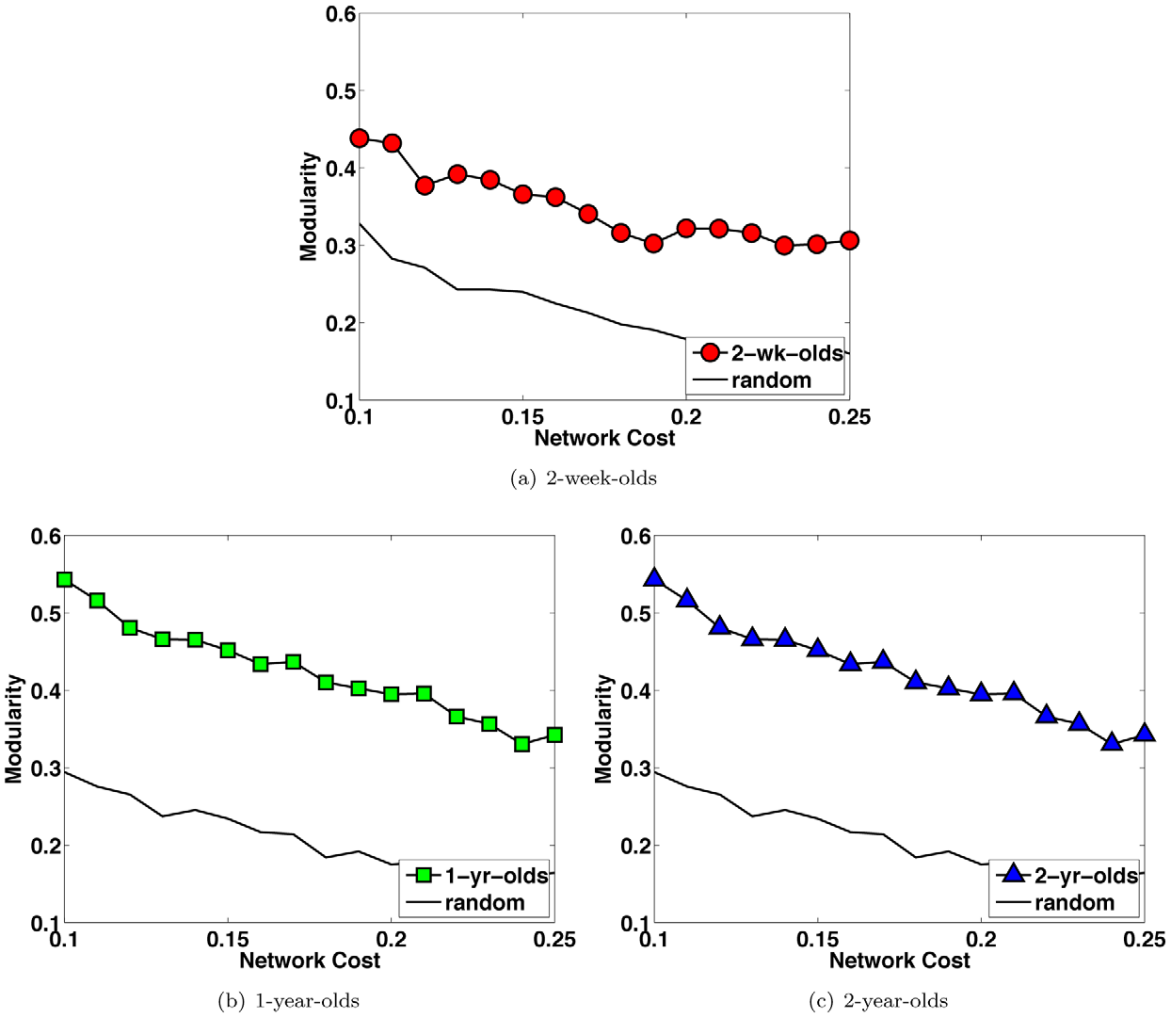
Nonrandom Modularity. Comparing the modularities [15], [16] of the brain networks with comparable random networks indicates non-random network modularity.

**Figure 8:**
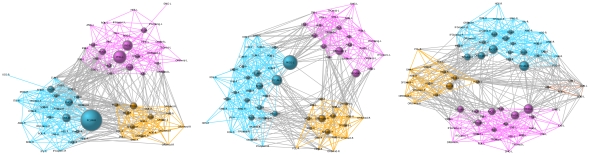
Network Communities. The spring-embedding visualization of networks is implemented with Kamada-Kawai layout algorithm using the Pajek [57] software package (pajek.imfm.si/doku.php). The nodes and intra-modular connections are colored-coded by the communities detected by the algorithm described in [16], while inter-modular connections are colored-coded with light-gray. The sizes of the vertices are weighted by the (logarithmically scaled) node betweenness [29]. Descriptions of the abbreviated region labels can be found in Table 1. See Table 3 for the constituent regions in each community.

Modularity [15], [16] of the early developing brain networks was analyzed over a range of diffusivity thresholds and compared with random networks. As shown in Fig. 7, the brain networks have consistently higher modularities than comparable random networks. For a better idea of how the brain network is organized, we detected brain network communities using a fast community detection algorithm that partitioned the network into subnetworks to achieve maximum network modularity [16]. The modularity metric quantifies how different intra-modular links in a network are from a random network with the same modular organization [15]. The results, shown in Fig. 8 (see Table 3 for the constituent regions in each community), indicate that the pediatric brain is organized into a number of internally densely connected subnetworks with sparser connections relating them to work as an organic whole.

**Table 3:** Constituent Regions in Each Community.

Age Group	Community	Regions
2-wk-olds	1	PreCG-L ORBsupb-L MFG-L ORBmid-L IFGoperc-L IFGtriang-L ORBinf-L ROL-L OLF-L ORBmed-L REC-L INS-L PHG-L CAL-L LING-L SOG-L MOG-L IOG-L FFG-L PoCG-L SPG-L IPL-L SMG-L ANG-L HES-L STG-L TPOsup-L MTG-L TPOmid-L ITG-L
	2	SFGdor-L SFGdor-R ORBsupb-R ORBmid-R SMA-L SMA-R OLF-R SFGmed-L SFGmed-R ORBmed-R REC-R ACG-L ACG-R MCG-L MCG-R PCUN-L PCL-L PCL-R
	3	PreCG-R MFG-R IFGoperc-R IFGtriang-R ORBinf-R ROL-R INS-R PCG-L PCG-R PHG-R CAL-R CUN-L CUN-R LING-R SOG-R MOG-R IOG-R FFG-R PoCG-R SPG-R IPL-R SMG-R ANG-R PCUN-R HES-R STG-R TPOsup-R MTG-R TPOmid-R ITG-R
1-yr-olds	1	PreCG-R SFGdor-R ORBsupb-R MFG-R ORBmid-R IFGoperc-R SMA-L SMA-R OLF-R SFGmed-L SFGmed-R ORBmed-L ORBmed-R REC-R ACG-L ACG-R MCG-L MCG-R PCL-L PCL-R
	2	PreCG-L SFGdor-L ORBsupb-L MFG-L ORBmid-L IFGoperc-L IFGtriang-L ORBinf-L ROL-L OLF-L REC-L INS-L PHG-L FFG-L PoCG-L IPL-L SMG-L ANG-L HES-L STG-L TPOsup-L MTG-L TPOmid-L ITG-L
	3	IFGtriang-R ORBinf-R ROL-R INS-R PCG-L PCG-R PHG-R CAL-L CAL-R CUN-L CUN-R LING-L LING-R SOG-L SOG-R MOG-L MOG-R IOG-L IOG-R FFG-R PoCG-R SPG-L SPG-R IPL-R SMG-R ANG-R PCUN-L PCUN-R HES-R STG-R TPOsup-R MTG-R TPOmid-R ITG-R
2-yr-olds	1	PreCG-L SFGdor-L ORBsupb-L MFG-L ORBmid-L IFGoperc-L IFGtriang-L ORBinf-L ROL-L OLF-L REC-L INS-L PHG-L LING-L MOG-L IOG-L FFG-L PoCG-L IPL-L SMG-L ANG-L HES-L STG-L TPOsup-L MTG-L TPOmid-L ITG-L
	2	PreCG-R SFGdor-R ORBsupb-R MFG-R ORBmid-R IFGoperc-R IFGtriang-R ORBinf-R ROL-R INS-R PCG-R PHG-R CAL-R CUN-R LING-R SOG-R MOG-R IOG-R FFG-R PoCG-R SPG-R IPL-R SMG-R ANG-R PCUN-R HES-R STG-R TPOsup-R MTG-R TPOmid-R ITG-R
	3	SMA-L SMA-R OLF-R SFGmed-L SFGmed-R ORBmed-L ORBmed-R REC-R ACG-L ACG-R MCG-L MCG-R PCL-L PCL-R
	4	PCG-L CAL-L CUN-L SOG-L SPG-L PCUN-L

**Figure 9:**
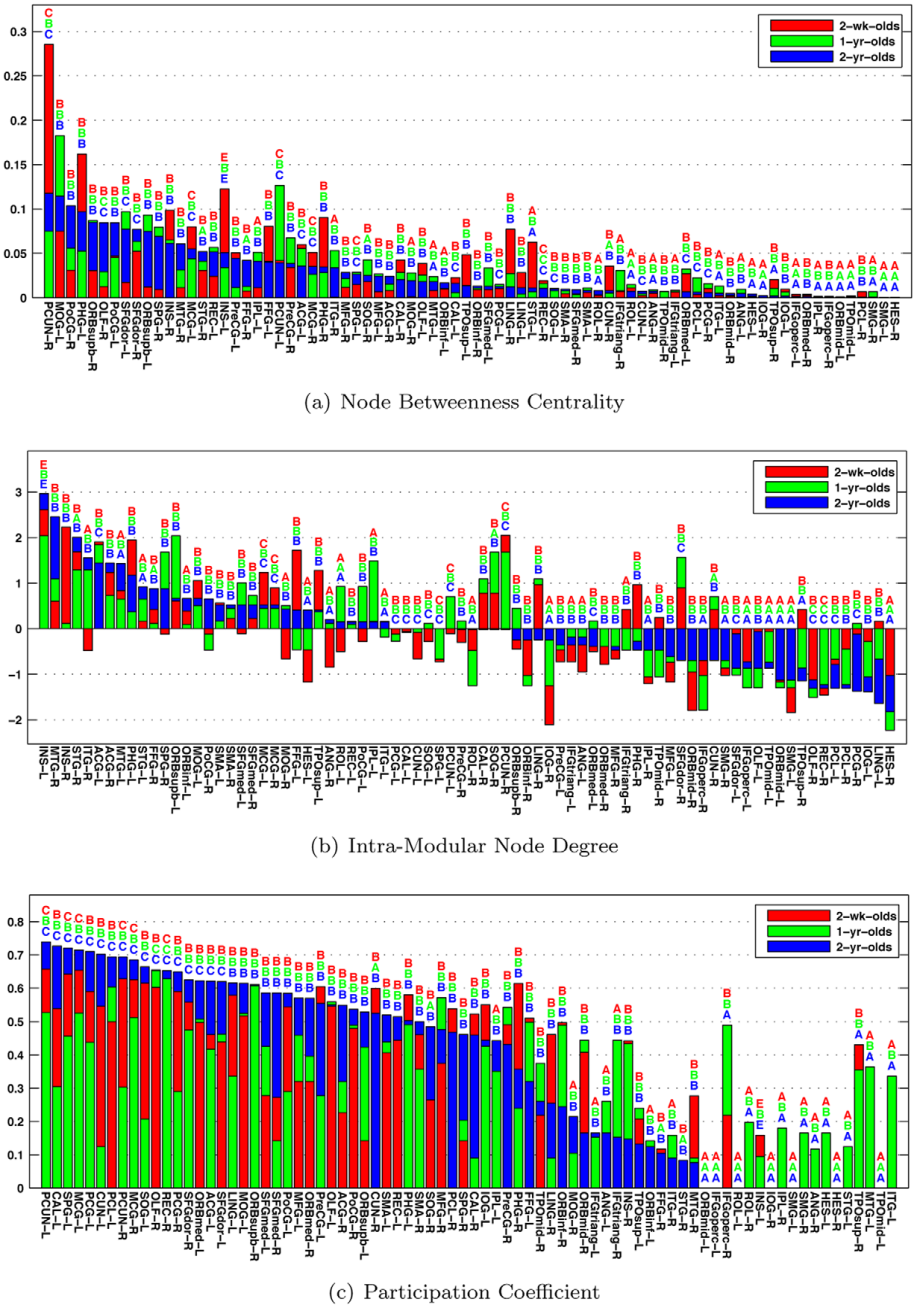
Betweenness Centrality, Intra-Modular Degree, and Participation Coefficient. The values are sorted based those of the 2-year-olds. The role of each node, as defined in [26], is specified above the respective bar: (A) non-hub ultra-peripheral node; (B) non-hub peripheral node; (C) non-hub connector nodes; and (D) non-hub kinless nodes; (E) provincial hubs; (F) connector hubs; and (G) kinless hubs. No node was found to satisfy the conditions required by (F) and (G).

To further study the role of each node, we computed the intra-modular degree and participation coefficient of each node. Following the approach in [26], nodes with 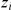 greater than 2.5 are classified as module hubs, otherwise classified as non-hubs. Non-hub nodes are divided into four different roles: (A) ultra-peripheral nodes; (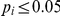); (B) peripheral nodes; that is, nodes with most links within their module (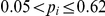); (C) non-hub connector nodes; that is, nodes with many links to other modules (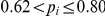); and (D) non-hub kinless nodes; that is, nodes with links homogeneously distributed among all modules (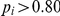). Hub nodes are divided into three different roles: (E) provincial hubs; that is, hub nodes with the vast majority of links within their module (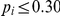); (F) connector hubs; that is, hubs with many links to most of the other modules (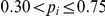); and (G) kinless hubs; that is, hubs with links homogeneously distributed among all modules (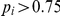). The results, shown in Fig. 9, indicate relatively large changes in nodal topological roles over age, reflecting dynamic brain developmental pattern in the first years of life.

Interestingly, the precuneus (PCUN), which shows high centrality and participation coefficient values, has been shown in previous literature to play an important role in the default mode network [42] and conciousness [43]. Its strategic location and wide-spread connections suggest that the precuneus is a major association area that may subserve a variety of behavioural functions [43]. Further investigation is needed for more detailed analysis of specific regions in relation to the dynamic growth patterns of the brain in the first years of life.

### Removal of High Betweenness Nodes Results in High Degree of Information Disruption

**Figure 10:**
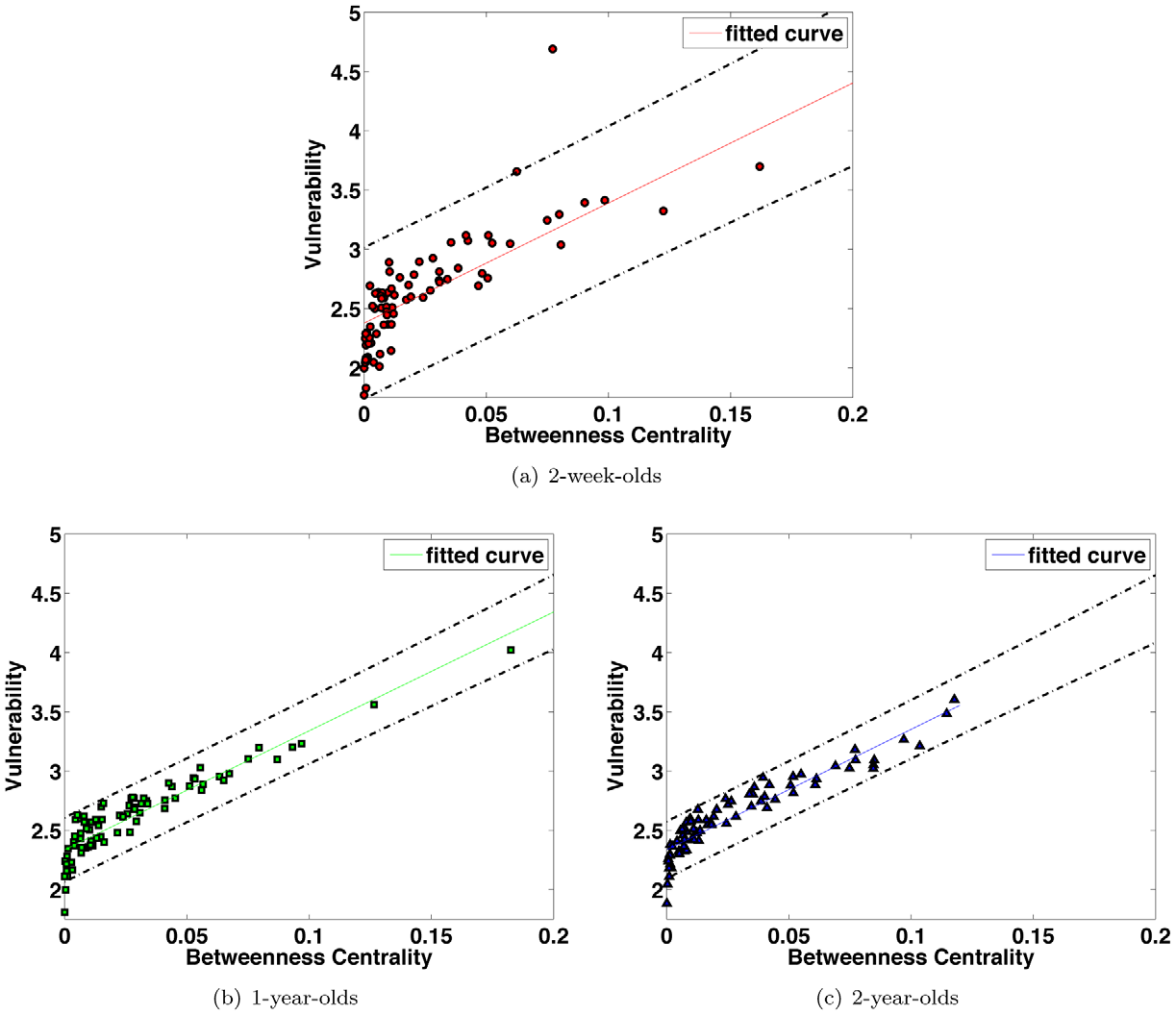
Betweenness Centrality and Vulnerability. Removal of a node with high betweenness generally results in a significant disruption of information flow in the brain network as indicated by a higher vulnerability value. The dashed lines indicate 95% confidence interval. The betweenness centrality value is normalized by division by the total number of possible connections 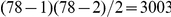.

Pathological development, related for instance to neonatal stroke, can be simulated by destroying some network nodes. This can be quantified by two measures: betweenness centrality and vulnerability. Betweenness centrality is a measure for gauging the importance of a node in the overall information flow, and network vulnerabiliy measures the disruption of information flow when a node is removed from the network. We found a linear correlation between the betweenness and vulnerability in each age groups, shown in Fig. 10, indicating the removal of a node with high betweenness centrality value will result in a high degree of connection loss in the brain network.

### Symmetry of Cerebral Hemispheres in the First Years of Life

**Figure 11:**
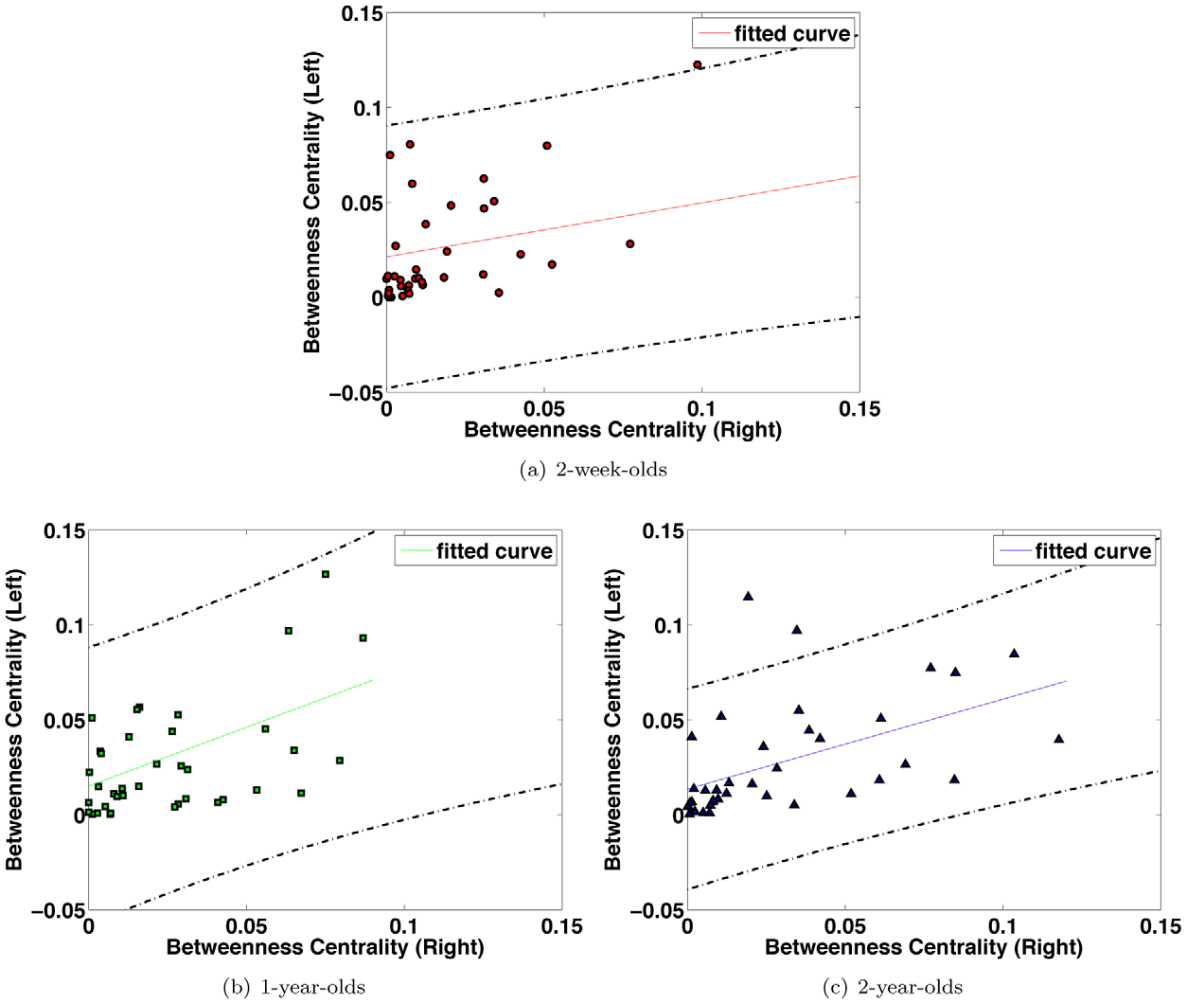
Inter-Hemispheric Correlation of Node Betweenness. Each circle gives the left and right betweenness value for each node. Each age group shows a rightward assymetry - indicated by the slope values 0.2843, 0.6202, and 0.4738, respectively (1 indicates perfect symmetry). The dashed lines indicate 95% confidence interval. The betweenness centrality value is normalized by division by 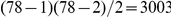.

Structural asymmetries of the human brain appear to underlie functional asymmetries. Cerebral asymmetries in the adult brain include the right hemisphere being larger than the left hemisphere, accounted for mainly by more white matter on the right [44]. Adult patterns of cerebral asymmetry have also been observed in older children [45]. For the pediatric subjects we studied, we found that the node betweenness of the left and right hemispheres were linearly correlated (Fig. 11) with an overall asymmetry towards the right hemisphere. The Pearson correlation coefficients of the left-right betweenness values for the three age groups are 0.3890 (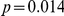), 0.4096 (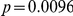), and 0.5113 (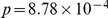), respectively. Cerebral symmetry appears to be less consistent for the 2-week-olds compared with the 1-year-olds and the 2-year-olds, judging from the slope and the goodness of fit: 0.2843 (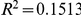), 0.6202 (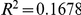), and 0.4738 (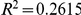).

### Sexual Dimorphism

Sexual dimorphism are present in the adult brain, with males having larger brain volumes [44], [46]. We studied how the pediatric male and female brains differ from the point of view of white-matter connectivity. Separating the subjects into male and female (18 female subjects were randomly selected to match the number of male subjects), we generated a backbone connectivity network for each gender-age group. We then computed the global and local efficiency for each of these networks. The results, as shown in Table 4, indicate that males generally have higher global and local efficiency compared with females.

**Table 4:** Network Efficiency of the Male and Female Brains.

	Global Efficiency			Local Efficiency		
	2-week-olds	1-year-olds	2-year-olds	2-week-olds	1-year-olds	2-year-olds
Male	0.5550	0.5626	0.5596	0.7574	0.7630	0.7828
Female	0.5673	0.5673	0.5673	0.7563	0.7563	0.7563
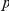 -value	0.277	0.281	0.008	0.949	0.621	0.002

## Discussion

Brain growth is not uniform: there is a differential growth between subcortical and cortical regions, and between different regions of the cortex. For example, there is a rapid burst of synapse formation in the visual cortex between 3 and 4 months, with the maximum density reached between 4 and 12 months. Synaptogenesis starts at the same time in the prefrontal cortex, but the synapse density increases much more slowly and does not reach its peak until well after the first year [47]. Therefore, one would expect that, in the course of development, there would be a remodeling of the interaction between brain regions.

This report employs a graph theoretic approach, which leverages connectivity information afforded by diffusion tensor imaging (DTI) fiber tractography, to examine the development of the brain network in the first years of life. We hope to gain deeper insights into the seldom studied critical period of human brain development, and accrue knowledge as the basis for understanding the nature of the adult brain.

A number of recent studies employ diffusion MRI for investigation of human brain anatomical networks, but none of them involves pediatric subjects. The first effort by Hagmann et al. [12] confirmed the small-world [24] nature of the anatomical networks of individual brains. The network nodes are defined in a subject-specific fashion at a fine-grained voxel level where the white-matter-grey-matter boundary is partitioned into thousands of ROIs. This approach, while allowing high resolution analysis of brain connectivity, makes comparison across subjects rather difficult owing to the requirement of high registration precision to match across subjects the small ROIs. The second study, conducted by Iturria-Medina et al. [14], models the brain using a weighted, instead of the commonly used unweighted, anatomical network. An Anatomical Connection Probability (ACP) matrix [48], which measures the maximum probability of any two regions to be connected at least by a single nervous fiber connection, is used to characterize the brain network. Under this framework, however, nonzero connection probabilities are assigned to many brain region pairs, even those, based on other studies, which are unlikely to be connected (e.g., left frontal and right occipital cortex). The most recent study was done by Gong et al. [13] and involves 80 subjects of 18–31 years old. Their aim is to establish a population-based anatomical network capturing the underlying common connectivity patterns of the cerebral cortex (i.e., backbone) across young healthy adults, rather than a subject-specific and very detailed network for an entire individual brain. Consistent with other studies, they reported that the cortical network exhibits a prominent small-world attribute. They also observed that the network has an exponentially truncated power-law topological distribution [39]. Gong and his colleagues [25] further extended their work to study the age- and gender-related differences in the cortical anatomical network.

### Broad-Scale Networks

A broad-scale network is characterized by a partial observation of the power-law before a sharp fall off in the node degree distribution. This indicates that the brain network includes some pivotal nodes (i.e., hubs) and edges (i.e., bridges) but prevents the existence of huge hubs or bridges with too much load. A broad-scale network is more resilient to targeted attack on its hubs than a comparable scale-free network [49], but about equally resilient to random error [8].

In the first years of life, the synaptic pruning process, which removes more than half of the synapses up to puberty [50], may perhaps defy the effect of the growth preferential attachment mechanism [49] needed to form a scale-free network [49]. Further support that the brain network is not scale-free is Achard et al.'s [8] observation that under the growth preferential attachment mechanism of a scale-free network, one would not expect the relatively late-developing regions such as the dorsolateral prefrontal cortex to be among the hubs of the network. In this respect, our observation is also consistent with Gong et al. [13], who demonstrated that similar network architecture can be found in matured subjects (18–31 years of age).

We should note here that, in contrast to our findings, Hagmann et al. [12] found that the node degrees exhibit a distribution with exponential tail. The authors further suggested that from a developmental point of view, hubs do not seem to be favored. Eguiluz et al. [51] found that the human brain functional network had a scale-free nature at a voxel level.

### The Small-World Nature of Pediatric Brain Networks

Although, from neurogenesis to myelination and gyrification, the brain undergoes significant changes, the overall adult brain patterns are present at 2 years of age [2]. It is hence not surprising that the 2-year-old brain network, consistent with previous studies [8], [9], [12]–[14], [51], is small-world in nature as indicated by its local and global efficiency. It is, however, important to note that the neonatal brain network also shows characteristic higher local efficiency than a comparable random network, and higher global efficiency compared to a regular lattice, albeit with lower efficiency compared to the other two age groups. Fig. 4 reveals that the lower efficiency of the neonatal brain network might be a result of the overall lower number of matured long fibers. This is perhaps not surprising as it is a known fact that myelination is happening rapidly during the first year of life, and the longer fibers might be still in their developmental stage. As the brain develops, neural fibers of farther reach begin to mature and hence results in higher efficiency.

### Local-to-Distributed Organization

It has been recently observed that from children to adults, the organization of the brain functional network shifts from a local anatomical emphasis to a more distributed architecture [36]. Our observation from the fiber tractography based analysis of the brain structural network provides direct supportive evidence for this hypothesis. We first note that Fig. 5, as previously analyzed, indicates an overall increase in maturation of fibers with longer lengths, especially during the first year of life. This implies that matured connections of the neonatal brain are predominantly short range, signifying anatomical proximity. As the brain develops, long range fibers also starts to matures. We note here that the change of fiber lengths as measured by the tractography algorithm by no means indicate that the change happens physically with the actual neuronal fibers. Post-mortem studies indicate that the fibers do not grow after birth and that axonal wiring between distant regions does not change. Therefore, the change of connection length should not be taken as changes in axonal length, but as changes in maturation processes.

### Gender Effects on Network Properties

It is interesting to observe from Table 4 that the global and local efficiency of the brain network begins to show significant gender differences at the age of two. Gender effects on network properties have been observed in recent related studies [25], [52], albeit on adult subjects. Of note, in contrast to the current study, the study in [25] observed greater local efficiency in female compared with male subjects. To resolve this discrepancy, subjects with age ranging from infancy to young adulthood need to be scanned and studied. Significant brain changes in this critical period of growth would alter the brain network and hence its measured properties. Studying and understanding brain structural changes in this period of time will shed new light on how the results from the current study and those in [25] could be bridged.

### Methodological Issues

DTI, while providing a convenient way of probing into the brain microstructures, suffers from the well-documented limitation of not being able to encode complex multi-directional diffusion patterns. Ideally, imaging techniques such as High Angular Resolution Diffusion Imaging (HARDI) [53] should be used. But the noise created by the EPI sequence makes scanning with a long duration difficult, especially pediatric subjects who wake up quite easily. Nevertheless, the availability of a set of HARDI data for pediatric subjects would surely be useful for a more precise deliniation of white matter connectivity in the first years of life.

Tractography depends on connection of pixel-by-pixel information and is hence sensitive to noise and pathway interruption by secondary causes such as anatomy of adjacent tracts, merging axons, and partial voluming. Probabilistic tractography [54] can potentially ameliorate these problems by providing confidence estimates of the reconstructed fiber trajectories. In [54] for instance, the fibers are reconstructed by sampling a Bayesian posterior distribution using a Markov Chain Monte Carlo (MCMC) approach, resulting in a large number of trajectories; some genuine and others inevitably spurious. The *probability* that a region is traversed by an anatomically genuine fiber bundle is then determined by normalizing the number of trajectories passing through that region with respect to the total number of trajectories initiated from the seed region. *Global* tractography methods [55], [56] reconstruct fiber trajectories by optimizing some global cost functions and are hence more robust to inaccuracy in the estimation of local fiber orientations, making them viable alternatives for overcoming problems associated with conventional tractography algorithms. Future work will be directed to evaluate how these different tractography algorithms affect the outcome of the analysis.

Both structural and functional based brain network analyses have been shown to provide valuable insights into the interactive mechanisms of different brain functional regions. While structural changes are more stable and more readily detectable, functional changes provide neuronal activation information which is often elusive structurally. Future work will hence hence be directed to employ a more comprehensive description of brain connectivity using information agglomerated from various imaging modalities such as T1-weighted, diffusion-weighted, and functional imaging.

### Concluding Remarks

To the best of our knowledge, this is the first report on the human brain structural connectivity quantified by fiber tractography involving pediatric subjects with longitudinal data. We employed a graph-theoretic approach to capture the common connectivity patterns of 39 pediatric subjects at 3 different time points (2 weeks, 1 year and 2 years). The networks exhibit small-world nature with node degree distributions indicating broad-scale characteristics. A study of the fiber length distributions indicate that the brain favors dense local connections over global long connections, consistent with the small-worldness nature of the brain network. The network evolution pattern over age gives supportive evidence for the brain local-to-distributed organizational trend, in line with the results obtained in previous studies performed on adult brains. We have also touched on issues such as network vulnerability, cerebral asymmetry and sexual dimorphism. Since structural growth underlies maturation of cognitive function, we believe that this structural-connectivity-based study is contributive to the better understanding of cognitive development.

## References

[1] Sporns O, Tononi G, Kötter R (2005). The human connectome: A structural description of the human brains.. PLoS Computational Biology.

[2] Gilmore JH, Lin W, Gerig G (2006). Fetal and neonatal brain development.. The American Journal of Psychiatry.

[3] Huang H, Zhang J, Wakana S, Zhang W, Ren T (2006). White and gray matter development in human fetal, newborn and pediatric brains.. Neuro Image.

[4] Achard S, Bullmore E (2007). Efficiency and cost of economical brain functional networks.. PLoS Computational Biology.

[5] Latora V, Marchiori M (2001). Efficient behavior of mall-world networks.. Physical Review Letters.

[6] Latora V, Marchiori M (2003). Economic small-world behavior in weighted networks.. European Physical Journal B.

[7] Salvador R, Suckling J, Coleman MR, Pickard JD, Menon D (2005). Neurophysiological architecture of functional magnetic resonance images of human brain.. Cerebral Cortex.

[8] Achard S, Salvador R, Whitcher B, Suckling J, Bullmore E (2006). A resilient, low-frequency, small-world human brain functional network with highly connected association cortical hubs.. The Journal of Neuroscience.

[9] Liu Y, Liang M, Zhou Y, He Y, Hao Y (2008). Disrupted small-world networks in schizophrenia.. Brain.

[10] Wang J, Wang L, Zang Y, Yang H, Tang H (2009). Parcellation-dependent small-world brain functional networks: A resting-state fMRI study.. Human Brain Mapping.

[11] He Y, Chen ZJ, Evans AC (2008). Structural insights into aberrant topological patterns of largescale cortical networks in alzheimers disease.. Journal of Neuroscience.

[12] Hagmann P, Kurant M, Gigandet X, Thiran P, Wedeen VJ (2007). Mapping human wholebrain structural networks with diffusion MRI.. PLoS ONE.

[13] Gong G, He Y, Concha L, Lebel C, Gross DW (2009). Mapping anatomical connectivity patterns of human cerebral cortex using in vivo diffusion tensor imaging tractography.. Cerebral Cortex.

[14] Iturria-Medina Y, Sotero RC, Canales-Rodríguez EJ, Alemán-Gómez Y, Melie-García L (2008). Studying the human brain anatomical network via diffusion weighted MRI and graph theory.. Neuroimage.

[15] Newman MEJ, Girvan M (2004). Finding and evaluating community structure in networks.. Physical Review E.

[16] Clauset A, Newman MEJ, Moore C (2004). Finding community structure in very large networks.. Physical Review E.

[17] Gilmore JH, Lin W, Prastawa MW, Looney CB, Vetsa YSK (2007). Regional gray matter growth, sexual dimorphism, and cerebral asymmetry in the neonatal brain.. Journal of Neuroscience.

[18] Basser PJ, Mattiello J, LeBihan D (1994). Estimation of the effective self-diffusion tensor from the NMR spin echo.. Journal of Magnetic Resonance Series B.

[19] Zhu H, Zhang HP, Ibrahim JG, Peterson BS (2007). Statistical analysis of diffusion tensors in diffusion-weighted magnetic resonance image data.. Journal of the American Statistical Association.

[20] Yap PT, Wu G, Zhu H, Lin W, Shen D (2009). Fast tensor image morphing for elastic registration.. MICCAI 2009 LNCS.

[21] Yap PT, Wu G, Zhu H, Lin W, Shen D (2010). F-TIMER: Fast Tensor Image Morphing for Elastic Registration.. IEEE Transactions on Medical Imaging.

[22] Leemans A, Jeurissen B, Sijbers J, Jones DK (2009). ExploreDTI: A graphical toolbox for processing,analyzing, and visualizing diffusion MR data.. in 17th Annual Meeting of Intl Soc Mag Reson Med.

[23] Tzourio-Mazoyer N, Landeau B, Papathanassiou D, Crivello F, Etard O (2002). Automated anatomical labeling of activations in SPM using a macroscopic anatomical parcellation of the MNI MRI single-subject brain.. Neuroimage.

[24] Watts DJ, Strogatz SH (1998). Collective dynamics of ‘small-world’ networks.. Nature.

[25] Gong G, Rosa-Neto P, Carbonell F, Chen ZJ, He Y (2009). Age- and gender-related differences in the cortical anatomical network.. Journal of Neuroscience.

[26] Guimerá R, Amaral LAN (2005). Functional cartography of complex metabolic networks.. Nature.

[27] Meunier D, Achard S, Morcom A, Bullmore E (2009). Age-related changes in modular organization of human brain functional networks.. NeuroImage.

[28] Newman MEJ (2005). A measure of betweenness centrality based on random walks.. Social Networks.

[29] Freeman LC (1977). A set of measures of centrality based on betweenness.. Sociometry.

[30] Costa LD, Rodrigues FA, Travieso G, Boas PRV (2007). Characterization of complex networks: a survey of measurements.. Adv Phys.

[31] Maslov S, Sneppen K (2002). Specificity and stability in topology of protein networks.. Science.

[32] Sporns O, Zwi JD (2004). The small world of the cerebral cortex.. Neuroinformatics.

[33] Nicoll A, Blakemore C (1993). Patterns of local connectivity in the neoncortex.. Neural Computation.

[34] Liley DTJ, Wright JJ (1994). Intracortical connectivity of pyramidal and stellate cells: estimate of synaptic densities and coupling symmetry.. Computation in Neural Systems.

[35] Chen BL, Hall DH, Chklovskii DB (2005). Wiring optimization can relate neuronal structure and function.. Proceedings of the National Academy of Sciences of the United States of America.

[36] Fair DA, Cohen AL, Power JD, Dosenbach NUF, Church JA (2009). Functional brain networks develop from a “local to distributed” organization.. PLoS Computational Biology.

[37] Fair DA, Dosenbach NNF, Church JA, Cohen AL, Brahmbhatt S (2007). Development of distinct control networks through segregation and integration.. PNAS.

[38] Kagen J, Herschkowitz N (2005). A young mind in a growing brain.

[39] Amaral LAN, Scala A, MBarthélémy, Stanley HE (2000). Classes of small-world networks.. PNAS.

[40] Albert R, Jeong H, Barabasi AL (2000). Error and attack tolerance of complex networks.. Nature.

[41] Strogatz SH (2001). Exploring complex networks.. Nature.

[42] Franssona P, Marrelec G (2008). The precuneus/posterior cingulate cortex plays a pivotal role in the default mode network: Evidence from a partial correlation network analysis.. Neuro Image.

[43] Cavanna AE, Trimble MR (2006). The precuneus: a review of its functional anatomy and behavioural correlates.. Brain.

[44] Nopoulos P, Flaum M, O'Leary D, Andreasen NC (2000). Sexual dimorphism in the human brain: evaluation of tissue volume, tissue composition and surface anatomy using magnetic resonance imaging.. Psychiatry Research.

[45] Herbert MR, Ziegler DA, Deutsch CK, O'Brien LM, Kennedy DN (2005). Brain asymmetries in autism and developmental language disorder: a nested whole-brain analysis.. Brain.

[46] Gur RC, Turetsky BI, Matsui M, Yan M, Bilker W (1999). Sex differences in brain gray and white matter in healthy young adults: Correlations with cognitive performance.. The Journal of Neuroscience.

[47] Johnson MH (2001). Functional brain development in humans.. Nature Reviews Neuroscience.

[48] Iturria-Medina Y, Canales-Rodríguez EJ, Melie-García L, Valdés-Hernández PA, Martínez-Montes E (2007). Characterizing brain anatomical connections using diffusion weighted MRI and graph theory.. Neuro Image.

[49] Barabási AL, Bonabeau E (2003). Scale-free networks.. Scientific American.

[50] Chechik G, Meilijson I, Ruppin E (1999). Neuronal regulation: A mechanism for efficient synaptic pruning during brain maturation.. Neural Computation.

[51] Eguíluz VM, Chialvo DR, Cecchi GA, Baliki M, Apkarian AV (2005). Scale-free brain functionalnetworks.. Physical Review Letters.

[52] Yan C, Gong G, Wang J, Wang D, Liu D (2010). Sex- and brain size-related small-world structural cortical networks in young adults: A dti tractography study.. Cerebral Cortex.

[53] Tuch DS, Weisskoff RM, Belliveau JW, Wedeen VJ (1999). High angular resolution diffusion imaging of the human brain..

[54] Friman O, Farnebäck G, Westin CF (2006). A bayesian approach for stochastic white matter tractography.. IEEE Transactions on Medical Imaging.

[55] Jbabdi S, Woolrich M, Anderson JLR, Behrens TEJ (2007). A bayesian framework for global tractography.. Neuro Image.

[56] Zalesky A (2008). DT-MRI fiber tracking: A shortest paths approach.. IEEE Transactions on Medical Imaging.

[57] Batagelj V, Mrvar A (1998). Pajek | program for large network analysis.. Connections.

